# A framework linking farming resilience with productivity: empirical validation from Poland in times of crises

**DOI:** 10.1007/s11625-021-01047-1

**Published:** 2021-10-11

**Authors:** Katarzyna Zawalińska, Adam Wąs, Paweł Kobus, Katarzyna Bańkowska

**Affiliations:** 1 Institute of Agricultural and Rural Development, Polish Academy of Sciences (IRWiR PAN), ul. Nowy Świat 72, 00-330 Warsaw, Poland; 2 Institute of Economics and Finance, Warsaw University of Life Sciences—SGGW, ul. Nowoursynowska 166, 02-787 Warsaw, Poland

**Keywords:** Resilience approaches, Agricultural total factor productivity, Green innovation, Ecosystem services, Global crises, Farming sectors

## Abstract

Farming sectors’ resilience has been built over decades with the aid of policies and institutions. However, its actual standing can be assessed in times of crises when farms have to overcome particular challenges. We use a large-scale farming sectors dataset FADN spanning 2006–2015 in which two major economic crises occurred—the global economic crisis of 2008 and the Russian embargo of 2014—to exemplify our approach to resilience’s assessment based on the Polish farming sectors. We introduce a distinction between “potential resilience” versus “revealed resilience” where the former is assessed based on resilience capacities (robustness, adaptability and transformability), while the latter is assessed based on the observed decomposition of total factor productivity (TFP) changes in response to the adverse economic shocks. Hence, the proposed framework directly links productivity with the two types of resilience. We applied the Färe-Primont method of TFP decomposition, into technological change and various types of efficiency changes and a detailed farm survey to distinguish between the drivers of technological changes in each farming sector such as specific innovations and ecosystem services. Our findings show that farms differ in their revealed resilience both among the sectors and between two different shock events. Only field crop farms and granivores farms (pig and poultry) maintained their resilience to both crises, staying robust and/or adaptable. The former had the most productive technology and were leaders in applying innovations while the latter were second best in innovations and fairly good in their application of ecosystem-based services into their technology. Other farm types failed to be resilient to the first crisis but proved robust during the second. The outcomes of the study have implications for sustainability oriented policies.

## Introduction

The link between total factor productivity (TFP) and resilience is not obvious. Generally, resilience is broadly defined as “[…] ability to ensure the provision of the system functions in the face of increasingly complex and accumulating economic, social, environmental and institutional shocks […]” (Meuwissen et al. 2019, p. 2). However, here, the notion of “specified resilience” also holds (O’Connell et al. 2016), that is resilience of particular parts of a system (in our case farming sectors which are part of farming systems) to identified disturbances, i.e. where their potential future occurrence is known or suspected (in our case economic crises), though the timing and magnitude may be unknown. The global positive trend of agricultural output driven by TFP growth shows that more agricultural output is produced with less input. It could be explained by production with less environmental externalities and positive feedbacks to ecosystem services, thus enhancing the resilience of farming sectors to challenges such as drought, diseases, political bans and market volatility, among others. Debates over the resilience and sustainability of farming and agricultural systems usually focus on the eco-socio-economic trade-offs. However, it is important to distinguish that resilience and sustainability are two different concept in many ways (Nagatsu et al. 2020; Shahadu 2016; Xu et al. 2015). Modern sustainability means more than just the ability to maintain the system but in particular encompasses three aspects: continuance, orientation, and relationships with other contemporaries, future generations, and nature (Becker 2012). In that sense, the sustainability subsumes resilience.

Related to the above trade-offs are notions of: “sustainable development”, i.e. “that is development that meets the needs of the present without compromising the ability of future generations to meet their own needs” (Brundtland et al. 1987); “wildlife-friendly” farming to promote biodiversity (Hardman et al. 2016; Quinn et al. 2012) and “sustainable intensification” to reduce the pressures of agriculture on ecosystems (Armstrong McKay et al. 2019; Scherer et al. 2018; Kumar et al. 2018; Rockström et al. 2017; Schiefer et al. 2016; Zhang et al. 2017; Ceddia et al. 2013; Garnett et al. 2013). However, the statistics show that most growth in global agricultural output, since the 1990s has come not from intensification or extensification but from more efficient use of labour, land, capital and inputs that boost the total factor productivity (TFP) of agriculture (Fuglie 2015; Fuglie et al. 2016).

In practice, we still do not know enough about it, since there is a paucity of empirical studies linking productivity and resilience explicitly in one consistent framework (Coomes et al. 2019). This is partly, because the standard TFP calculations do not include environmental and social outcomes, so they seem to not directly refer to resilience and sustainability. This is despite the fact that several attempts have been made to enhance TFP indicators to grasp aspects of environment and public goods (Melfou et al. 2007; Fuglie et al. 2016). Additionally, research on eco-efficiency—that is creation of more goods and services with fewer resources while producing less waste and pollution—is addressing this issue. The recent studies on this notion and its application to Poland can be found in Czyżewski et al. 2020a, Czyżewski et al. 2020b, Sulewski et al. (2020) and Grzelak et al. (2019).

Research carried out by OECD introduced the so-called total resource productivity index (TRP) which covers ecosystem services and non-market environmental goods under the umbrella of “environmentally adjusted TFP” (OECD 2011). There are also studies aiming at developing strategies enhancing productivity and environmental performance at the same time for overall socio-economic development—so-called green productivity growth and green total factor productivity (Peng 2020; Wang et al. 2020; Beltrán-Esteve et al. 2019).

It is important to realise that it is the TFP dynamics that interact with resilience, so the change in TFP matters rather than the absolute value of it, and that the links are in two directions (Coomes et al. 2019). First, only resilient and sustainable farming can result in TFP growth. Second, in cases of strong shocks resulting in a negative impact on productivity, resilience and sustainability are also affected. Since the resilience and sustainability are two different notions, so also their links and interactions with TFP differ. We also separate them in our approach and focus on the former while the interrelations with the latter remain to be investigated within our framework in the future.

Thee resilience issues arise when adverse shocks have negative impacts on productivity and farm viability (Meuwissen et al. 2019), in our study, we observe the performance of farming sectors during the period 2006–2015 during which two major crises occurred. The first was in 2008, when all farm types in Poland were affected by an economic shock due to the worldwide financial crisis; this was transferred to the farming systems through: a severe impact on input and output prices, credit crunches, consumer behaviours and many other channels (Parlińska and Wielechowski 2009). The second was in 2014 (8th of August) when most farming sectors were affected by political shock due to the immediate, unexpected introduction of the Russian Embargo on most of the agri-food products from the EU to Russia. It had strong economic consequences on European agricultural products; the embargo constituted 46.3% of the agri-food export, i.e. 5.5 billion EUR. For Poland, the banned products covered 70% of the value of agri-food exports to Russia and most severely affected fruit, vegetables, milk and its associated processed products, as well as meat and its products (Kraciński, 2015; Rosińska-Bukowska, 2015). Hence, in our analyses, we especially investigate the changes in the resilience of the farming sectors around the time of these two shocks.

To bring more empirical depth into the theoretical discussion on links between TFP and resilience, we propose a distinction between “potential resilience” versus “revealed resilience”. This was conducted by analogy to the studies on competitiveness which became better understood after distinguishing between “potential” vs “revealed” competitiveness as proposed by David Ricardo. Similarly, in our framework, we propose a distinction between “potential resilience” measured by resilience capacities (robustness, adaptability and transformability), as proposed by Meuwissen et al. (2019), while the “revealed resilience” is measured ex-post the shocks, and in our case, theses are economic shocks so it is measured by observed TFP changes calculated by means of Färe-Primont indices (Färe and Primont, 1995). The two approaches in our framework are interlinked so it is possible to assess which potential resilience capacities result in actually revealed resilience.

According to the literature’s understanding, the dynamics of TFP growth is key in understanding how productivity can enhance or reduce agricultural resilience (Chavas, 2015). We add to this finding that the composition of this dynamic is even more important as it demonstrates the channels through which the resource saving is transmitted into agricultural production. From several approaches to TFP decomposition (Färe et al. 1994; Bureau et al. 1995; Brümmer et al. 2002; Coelli and Rao 2005; O’Donnell, 2010; Dakpo et al. 2016), we chose the Färe-Primont index decomposition approach as the most suitable for our purposes, explained in the methodological section.

Hence, the main goal of the paper is to compare the resilience of various farm types in Poland, when they experienced global shocks, with a focus on differences in their sources of technological change driving their TFP. This topic seems especially relevant now when the sectors are experiencing another global shock due to the COVID-19 pandemic. Hence understanding the type of resilience of each farming sector in the past facilitates the better targeting of policy actions in the future.

The added value of this paper is both theoretical and empirical. First, we proposed a novel distinction between potential and revealed resilience and put them into the consistent framework. Second, we linked recent studies on the resilience of farming systems with studies on TFP changes and its decomposition to grasp interlinkages between the two. Third, we empirically assessed the revealed resilience to economic shocks based on changes in TFP decomposition into detailed technological and efficiency changes by the farm types.

The paper is structured as follows. Section 2 provides a background on agricultural productivity over different economic regimes in Poland (communism, transition and membership in EU) and a description of our proposed framework linking productivity with resilience and sustainability. Section 3 presents the methods applied and the data acquired. Section 4 presents the results of the quantitative and qualitative analyses, while Sect. 5 discusses the meaning of the results in light of our framework and results of other studies. Section 6 concludes and suggests further avenues for future research.

## Background

### Total factor productivity as a driving force of future agricultural production

**Fig. 1:**
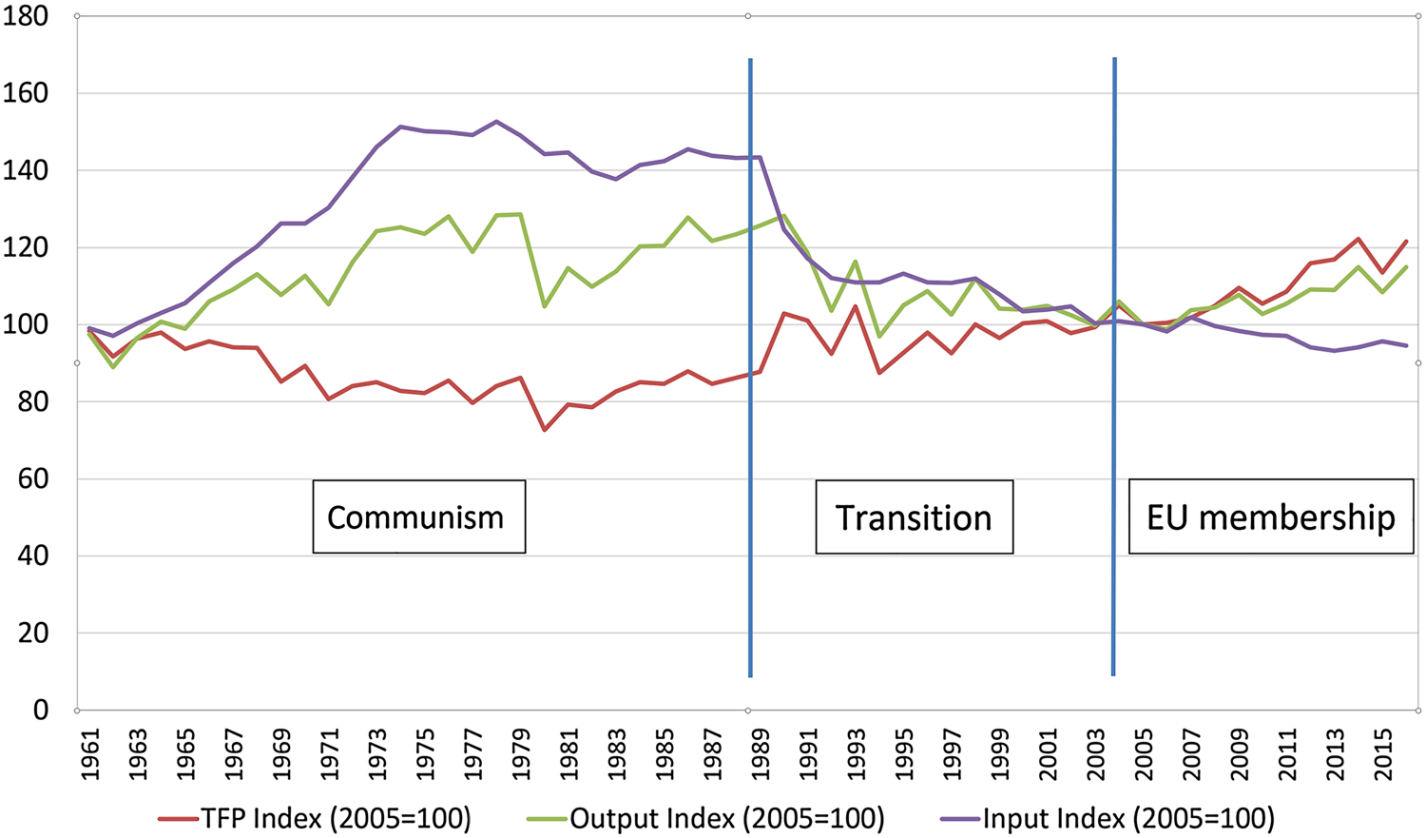
TFP, agricultural output and input changes in the Polish agriculture over different economic regimes during 1961–2015. Source: Own calculations based on (USDA, 2020)

In the ‘60 s, the output of agricultural production was mainly driven by input intensification, so more inputs were used to produce higher output. However, this was not sustainable in the long run and in the ‘70 s the global agricultural output began to be driven by TFP. This means that the global agricultural production increases with simultaneous reductions in the use of agricultural inputs (Coomes et al. 2019). Components of this TFP growth by definition are enhancements of technological change and an increased efficiency of inputs use, where it is argued that the former is more likely to be the main driver between the two (Dakpo et al. 2016). At the same time, the development of TFP and agricultural output in Poland went through three fundamentally different economic regimes: communism, transformation and EU membership—see Fig. 1. It is quite clear that in the communistic time (50 s–80 s) in the centrally planned economy the agricultural output was kept at an artificial level due to high use of inputs heavily supported by the state, while the TFP was declining most of the time. This was clearly an inefficient method of economic performance which was unsustainable in the long run and collapsed in 1989. Then, in the time of transition to the market economy (1989–2004), the input support was suddenly removed so it declined drastically and hence so did agricultural output, because the sector was not yet prepared for the effective use of inputs. However, the farming sectors were learning a new approach so after a few years of disturbances TFP started growing, slowly but steadily. After joining the EU in 2004, the development of productivity in the agricultural sector in Poland started to resemble global trends. Agricultural output then started to increase and was driven by TFP, despite a further decline in the input use—Fig. 1.

### Proposed framework linking total factor productivity with resilience and sustainability

**Fig. 2:**
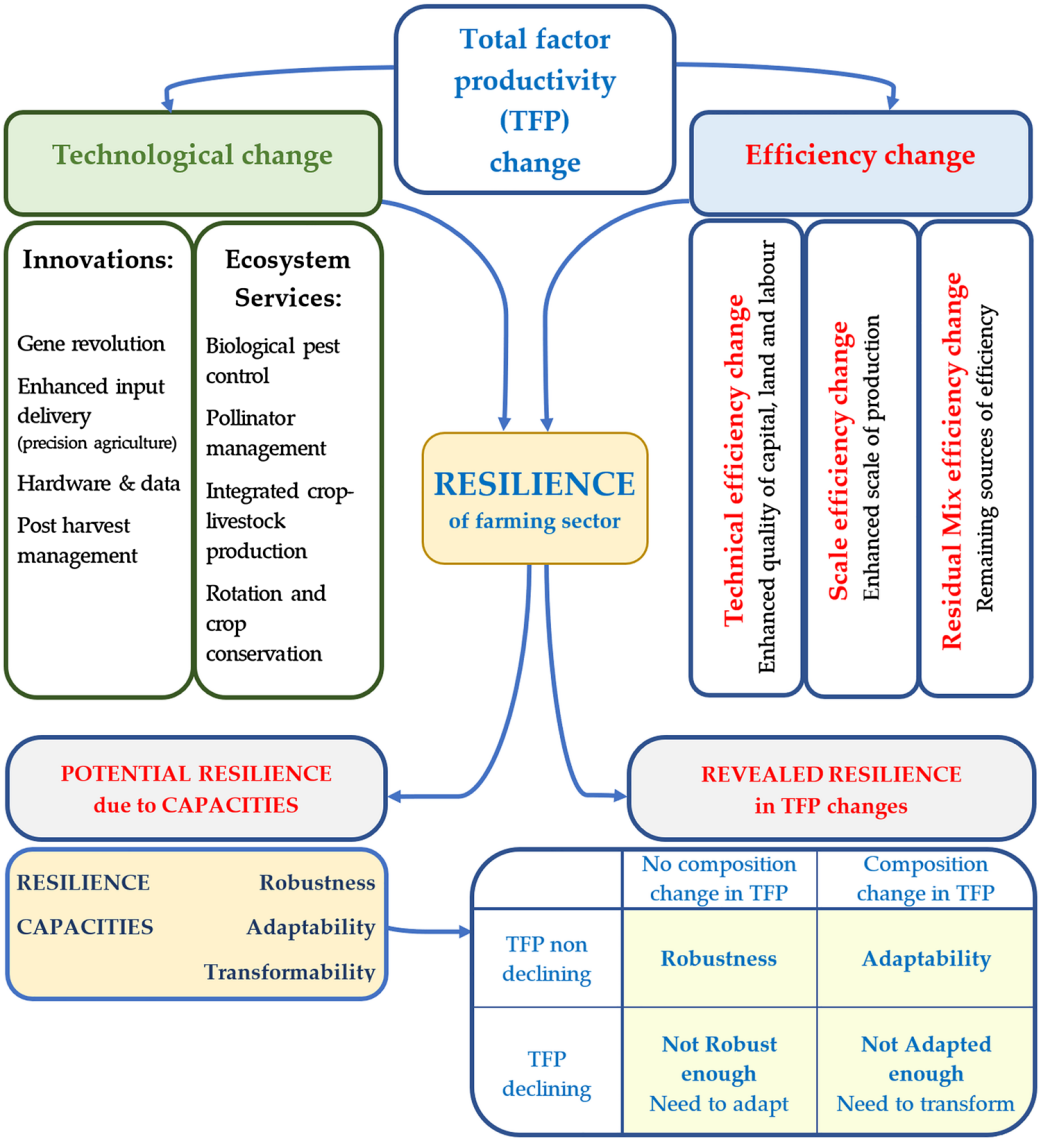
Framework linking total factor productivity dynamics with the resilience of farming sectors. Source: Own elaboration; Note: Robustness, Adaptability and Transformability are defined later in the Sect. Proposed framework linking total factor productivity with resilence and sustainability

Our framework brings in the concept which arose independently in recent literature on resilience and productivity, then links them by introducing a new distinction between “potential resilience” and “revealed resilience”—see the framework in Fig. 2. Studies on TFP dynamics have evolved and developed in such a direction as to allow researchers to distinguish with higher precision the drivers of TFP changes (Dakpo et al. 2018). This further allows researchers to distinguish between which parts of TFP dynamics come from technological changes versus efficiency changes—see the top of the framework figure. This is a crucial source of information from the point of view of resilience as it indicates that it is the productivity dynamics that interact with the resilience and sustainability (Chavas 2015). The TFP change decomposition—into technological change and three types of efficiency changes depicted in our framework in the upper part of Fig. 2—is formally described by Eqs. 1–7 in Sect. 3 on methods and data.

Recent studies suggest that technological changes contribute to resilience and sustainability not only through innovations but also through the ecosystem services adopted by farms (Coomes et al. 2019; Tittonell 2020). Following these studies, technological change can come on one side from innovations such as: gene revolution, enhanced input delivery, hardware and data, and post-harvest management. On the other hand, it can come from the application of ecosystem services such as: biological pest control, pollinator management, integrated crop-livestock practices, and rotation and soil conservation—see the top left section of Fig. 2. That is in line with discourse on “green innovations” (Peng 2020; Wang et al. 2020; Gupta and Barua 2018).

Resilience, as adopted in this study, is a concept developed based on adaptive cycles (Holling, 2001). We had to narrow our approach for the sake of applying our analysis, however, we are aware of many other approaches to resilience (Xu and Kajikawa, 2018; Shahadu, 2016; Jarzebski et al. 2016). In our approach, the resilience describes the capacity of systems to withstand adverse shocks and to recover, while maintaining their essential structure and functions (Walker et al. 2004; Folke, 2006). On one hand, resilient farming enhances TFP growth that is manifested in the ability to generate technological change and/or efficiency change. On the other hand, productive farming system management influences ecosystem services and natural capital of the farming system through externalities and feedbacks so in this way the TFP growth pathways affect resilience.

In our paper, we focus on the issue of how resilience is revealed in TFP changes and its decomposition. We distinguish, as explained in the introduction, between “potential resilience”—manifested in resilience capacities—and “revealed resilience”—manifested in TFP changes and its decomposition. Here, resilience capacities refer to the capacity of farming sectors to cope (robustness) and respond (adaptability and transformability) to shocks and stresses (Meuwissen et al. 2019). Hence, the farming sectors in our framework are potentially resilient due to three capacities:

- Robustness: this is the ability to maintain the basic functions of the system without major changes to its internal components (including attributes) and processes, despite the presence of external disturbances (Urruty et al. 2016).
- Adaptability: the ability of the system to adapt internal elements and processes in response to changing external circumstances and thus continue to develop along the previous trajectory while maintaining all vital functions (Folke et al. 2010).
- Transformability: the ability of a system to develop or incorporate new elements and processes to an extent that alters operational logic to maintain important functions (provision of private and public goods) when structural changes in the ecological, economic or social environment render the existing system unsustainable or dysfunctional (Walker et al. 2004). However, the functions may change over time, e.g. due to changing societal preferences (Meuwissen et al. 2019). Therefore, in fact, transformability is the capacity for a system to be transformed to a different system, which can actually change its identity (O’Connell et al. 2016).

We link the potential and revealed resilience in our framework by observing TFP changes (weather it declines, grows or stay the same) due to economic shocks and by assessing TFP change decomposition (whether it shows a withstanding approach or any kind of adaptation)—see bottom section of Fig. 2. Thereafter, we can assess the resilience of the farming sector system as follows:

- The system is robust if the decomposition of its TFP shows no substantial changes. This means that the system maintained its performance by withstanding the situation, meaning that technological change and efficiency components are maintained in similar proportions—while TFP is non-declining (stays the same or grows).
- The system is adaptable if the decomposition of its TFP shows substantial changes; technological change and efficiency components have substantially different contributions to TFP change, e.g. TPF driven by technological change becomes driven by efficiency change—while TPF is non-declining. In such cases, the system adapted its TFP.
- In the case of a declining TFP path, the system fails to be robust if there is no change in TFP composition so it can improve its resilience by adapting. If the system adapts and changes its TFP composition yet the TFP still declines, a larger adaptation is needed leading possibly to transformation.

The last part of the framework (bottom left section of Fig. 2) indicates that the resilience can also interact with sustainability (Coomes et al. 2019; Marchese et al. 2018; Xu and Kajikawa 2018; Dile et al. 2013). In our framework, the influence can be both through potential and revealed resilience. In the case of the former, it is because long-term robustness, adaptability and transformability aim to sustain the system. In the latter case, this is impossible for any system to sustain in the long-run without being productive—as is the experience of centrally planned economies which tried to do this and failed, as exemplified by the Polish case explained in Sect. Proposed framework linking total factor productivity with resilience and sustainability and depicted in Fig. 1. However, the details of the transmission channels of interactions between the two types of resilience on sustainability goes beyond the scope of this research.

## Methods and data

We chose the Färe-Primont index for our analyses as most appropriate due to the following advantages. First, it can be used for multilateral and multi-temporal comparisons, as we need here, because it is not based on price but quantity indices. That is important also because the crises we analyse always affect the prices so price-based index of TFP would be biased. Second, this index, in contrast to the Laspeyers, Paasche, Fisher and Tornquist and Malmquist indexes, passes the transitivity property which makes it superior for comparative analyses. Third, it is also superior to the others with respect to the clear division of productivity driven by technological change vs efficiency changes and the further detailed decomposition of efficiency changes into technical efficiency change, scale efficiency change and residual mix efficiency change. At the same time, the Malmquist index ignores the efficiency changes coming from the input/output mix. In our case, the clear decomposition of TPF changes is key, because the differences in resilience among the farm types in our framework are assessed based on the changes in the composition of those elements over time (i.e. no major changes in the decomposition indicates robustness, while other changes indicate either adaptability or transformability of the farming sector as explained in detail in our framework). Besides, knowing the contribution of technological changes driving TFP allows us to interpret, as suggested by Coomes et al. (2019), the importance of its drivers in division by those stemming from innovations (categorised into: gene revolution, enhanced input delivery, hardware and data, and post-harvest management) and/or ecosystem services (categorised into: biological pest control, pollinator management, integrated crop-livestock practices, and rotation and soil conservation). Consequently, it enables us to include information on particular technologies and ecosystem services from our farm survey distinguishing among five types of farming (field crop, horticulture, granivores, cattle, and mixed farms) to enrich the information on the drivers of technological change in TFP decomposition in each farm type.

### Färe-Primont index and its decomposition

For a given number (*N*) of decision-making units (DMUs), in our case individual farms, observed in the time from t to t + 1, each of them uses a certain vector of inputs x^t^_nk_ = (x^t^_n1,_ …, x^t^_nK_)’, where $x\epsilon {\mathbb{R}}_{+}^{K}$, to produce a certain vector of outputs y^t^_nq_ = (y^t^_n1_, …, y^t^_nQ_)’, where $y\epsilon {\mathbb{R}}_{+}^{Q}$. Then the benchmark production technology in period t is defined as:

1 $$\psi_{t} = \left[ {\left( {x^{t} ,y^{t} } \right) \in {\mathbb{R}}_{ + }^{K + Q} \,\,\,\,\,\left| {x^{t} \,{\text{can}}\,{\text{produce}}\,y^{t} } \right.} \right]$$

${\Psi }_{t}$ satisfies the standard conditions discussed in Chambers (1998), Fare (1988) and Fare and Grosskopf (1996, 2004).

The Färe-Primont productivity change index (FPP) for a DMU between time t and t + 1 is expressed as a product of: (1) technological change and (2) efficiency change:

2 $${\text{FPP}}_{t, t + 1} = \frac{{{\text{TFP}}_{t + 1} }}{{{\text{TFP}}_{t} }} = \frac{{{\text{TFP}}_{t + 1}^{*} }}{{{\text{TFP}}_{t}^{*} }}*\frac{{{\text{TFPE}}_{t + 1} }}{{{\text{TFPE}}_{t} }} = {\text{TC}}_{t, t + 1} *{\text{EC}}_{t, t + 1}$$

where total factor productivity ${\text{TFP}}_{t}$ is defined as a ratio of aggregate level of outputs Y(y^t^) to aggregated inputs X(x^t^) in time t (analogically ${\text{TFP}}_{t + 1}$, for t + 1); the first term on the right-hand side ${\text{TFP}}_{t + 1}^{*} /{\text{TFP}}_{t}^{*}$ is a measure of technological change (named ${\text{TC}}_{t, t + 1}$), and measures the difference between the maximum TFP using the maximum possible technology at period *t* and the maximum TFP possible at period t + 1—it is graphically expressed as a shift in the production frontier; the DMU experiences technological progress when the expression is greater than 1 or regresses when it is less than 1, or encounters no change when it is equal to 1. The technological change happens as a global phenomenon caused by changes in technology or factors affecting fundamental economic conditions. The analysed shocks were very substantial and world-wide so we assume these global factors are the same or similar for all our DMUs and, therefore, a shift in technology can be represented by the same frontier for all DMUs.

The second term in Eq. 2, ${\text{TFPE}}_{t + 1} /{\text{TFPE}}_{t}$ captures efficiency change (named ${\text{EC}}_{t, t + 1}$), which is a change in a distance from the frontier, since ${\text{TFPE}}_{t} = {\text{TFP}}_{t} /{\text{TFP}}_{t}^{*}$ is the ratio between observed and maximum productivity. This component of the Färe-Primont index can be further decomposed into the product of three elements—technical efficiency change, scale efficiency change and residual mix efficiency change. Usually, the decomposition is written separately for input- vs. output-oriented productivity changes (O’Donnell 2010), however, Dakpo et al. (2016) proposes an expression to account for both orientations simultaneously, which in practice is a geometric mean, as follows:

3 $${\text{EC}}_{t, t + 1} = \frac{{\sqrt {{\text{OTE}}_{t + 1} *{\text{ITE}}_{t + 1} } }}{{\sqrt {{\text{OTE}}_{t} *ITE_{t} } }}*\frac{{\sqrt {{\text{OSE}}_{t + 1} *{\text{ISE}}_{t + 1} } }}{{\sqrt {{\text{OSE}}_{t} *{\text{ISE}}_{t} } }}*\frac{{{\text{RME}}_{t + 1} }}{{{\text{RME}}_{t} }} = {\text{TEC}}_{t,t + 1} *{\text{SEC}}_{t,t + 1} *{\text{RME}}_{t,t + 1}$$

where the first component—technical efficiency change (${\text{TEC}}_{t,t + 1}$)—is expressed as ${{\sqrt {{\text{OTE}}_{t + 1} *{\text{ITE}}_{t + 1} } } \mathord{\left/ {\vphantom {{\sqrt {{\text{OTE}}_{t + 1} *{\text{ITE}}_{t + 1} } } {\sqrt {{\text{OTE}}_{t + 1} *{\text{ITE}}_{t + 1} } }}} \right. \kern-\nulldelimiterspace} {\sqrt {{\text{OTE}}_{t + 1} *{\text{ITE}}_{t + 1} } }}$ where OTE is output oriented technical efficiency (and ITE the input oriented one) and measures the maximum achievable total factor productivity with the use of the same aggregated amount of inputs (or outputs), while holding the input and output mixes fixed; The second component in Eq. 3—scale efficiency change (${\text{SEC}}_{t,t + 1}$)—is expressed as ${{\sqrt {{\text{OSE}}_{t + 1} *{\text{ISE}}_{t + 1} } } \mathord{\left/ {\vphantom {{\sqrt {{\text{OSE}}_{t + 1} *{\text{ISE}}_{t + 1} } } {\sqrt {{\text{OSE}}_{t + 1} *{\text{ISE}}_{t + 1} } }}} \right. \kern-\nulldelimiterspace} {\sqrt {{\text{OSE}}_{t + 1} *{\text{ISE}}_{t + 1} } }}$ where OSE is output scale efficiency and ISE is input scale efficiency. OSE is a ratio of OTE scores under constant returns to scale (CRS) versus variable returns to scale (VRS). As indicated by Dakpo et al. (2016), therefore, OSE captures the difference between TFP at a technically efficient point and the maximum possible TFP at the point of the mix-invariant optimal scale associated with the CRS mix-invariant production frontier; the third component of efficiency change in Eq. 3—residual mix efficiency change $\left( {{\text{RME}}_{t,t + 1} } \right)$—is expressed as (${{{\text{RME}}_{t,t + 1} } \mathord{\left/ {\vphantom {{{\text{RME}}_{t,t + 1} } {{\text{RME}}_{t} }}} \right. \kern-\nulldelimiterspace} {{\text{RME}}_{t} }}$), where RME captures the difference between TFP at a point located on the CRS mix-invariant production frontier and the maximum achievable total factor productivity TFP*.

It should be noted that the aforementioned decomposition is only one possibility, although it is the most suitable for our purposes. However, there is also the possibility to decompose the Färe-Primont index into other components, e.g. technical efficiency change, mix efficiency change and residual scale efficiency change (see Dakpo et al. 2018; O’Donnell 2010).

### Meta-frontier Färe-Primont index

In our analyses, DMUs belong to different farming types (field crops, horticulture, cattle, granivores, mixed farms and other) so it is reasonable to believe that they have distinct technologies. In that case, as suggested by Dakpo et al. (2016), it is also appropriate to estimate a meta-technology which would grasp all groups’ technologies (O’Donnell et al. 2008; Battese et al. 2004; Battese and Rao 2002). This approach facilitates the calculation of the technology gap ratio (TGR) which measures the difference between each group frontier and meta-frontier and assesses which groups are leading in shifting the meta-frontier. At the same time, TGR indicates the possible scope for improvement in the performance of each group if all DMUs in the group have the same access to the technologies of all other groups. This is an assumption of the meta-frontier approach which allows us to specify in time t for s different available technologies ranging within 1,…S the following meta-technology: $M_{t} = {\Psi }_{t}^{1} \cup {\Psi }_{t}^{2} \cup \ldots \cup {\Psi }_{t}^{s}$ where ${\Psi }_{t}^{s}$ is the benchmark technology of each group s defined as:

$\psi_{{_{t} }}^{s} = \left[ {\left( {x_{s}^{t} ,y_{s}^{t} } \right) \in {\mathbb{R}}_{ + }^{K + Q} \,\,\,\,\,\left| {x_{s}^{t} \,{\text{can}}\,{\text{produce}}\,y_{s}^{t} } \right.} \right]$ and $M_{t} = \left[ {\left( {x^{t} ,y^{t} } \right) \in {\mathbb{R}}_{ + }^{K + Q} \,\,\,\,\,\left| {x^{t} {\text{can}}\,{\text{produce}}\,y^{t} } \right.} \right]$. $M_{t}$ is defined here independently of each group of DMUs.

The meta-frontier Färe-Primont index ($$MFPP)$$ for the global technology enveloping all individual technologies between time t and t + 1 is computed as follows:

4 $${\text{MFPP}}_{t,t + 1} = \frac{{{\text{MTFP}}_{t + 1} }}{{{\text{MTFP}}_{t} }} = \frac{{{\text{MTFP}}_{t + 1}^{ * } }}{{{\text{MTFP}}_{t}^{ * } }} * \frac{{{\text{MTFPE}}_{t + 1} }}{{{\text{MTFPE}}_{t} }} = {\text{MTC}}_{t,t + 1} * {\text{MEC}}_{t,t + 1}$$

where analogically to Eq. 2, ${\text{MTC}}_{t, t + 1}$ is a meta-frontier technological change one for all DMUs and ${\text{MEC}}_{t, t + 1}$ is meta-frontier efficiency change for them.

Comparing the points of maximum productivity on the individual group frontiers (for each farm type) with that of the meta-frontier (for all farm types together), we obtain TGR as suggested by O’Donnell and Fallah-Fini, (2011), Dakpo et al. (2016); and Dakpo et al. (2018).

5 $${\text{TGR}}_{t}^{s} = \frac{{{\text{TFP}}_{t}^{*s} }}{{{\text{MTFP}}_{t}^{*} }}$$

where ${\text{TGR}}_{t}^{s}$ is the meta-technology ratio for group s in period *t*; ${\text{TFP}}_{t}^{*s}$ is the point of maximum productivity relative to the group’s frontier, and ${\text{MTFP}}_{t}^{*}$ is the meta-frontier point of maximum productivity.

Therefore finally, the MFPP index can be expressed for groups within time t and t + 1, with explicit encountering for TGR and its change (TGRC), as follows:

6 $${\text{MFPP}}_{t, t + 1} = \frac{{{\text{TGR}}_{t + 1}^{s} }}{{{\text{TGR}}_{t}^{s} }}*\frac{{{\text{TFPE}}_{t + 1}^{s} }}{{{\text{TFPE}}_{t}^{s} }} = {\text{TGRC}}_{t, t + 1}^{s} + {\text{TFPE}}_{t, t + 1}^{s}$$

where the first component is TGR change (${\text{TGRC}}_{t, t + 1}^{s}$)—expressed through the comparison of TGRs in two periods: $TGR_{t + 1}^{s} /TGR_{t}^{s}$—and the second component is meta-frontier TFP efficiency change ($TFPE_{t, t + 1}^{s}$) where $TFPE_{t}^{s}$ = $TFP_{t} /TFP_{t}^{*s}$ (analogically is calculated as $TFPE_{t + 1}^{s}$).

The meta-frontier TFP efficiency change can be further decomposed as in the case of individual TFP efficiency change index (analogically to Eq. 3), so the final decomposition of the MFPP has four components:

7 $${\text{MFPP}}_{t, t + 1} = {\text{TGRC}}_{t, t + 1}^{s} *{\text{MTEC}}_{t, t + 1}^{s} *{\text{MSEC}}_{t, t + 1}^{s} {\text{*MRME}}_{t, t + 1}^{s}$$

where meta-frontier TGR change (${\text{TGRC}}_{t, t + 1}^{s}$), meta-frontier technical efficiency change (${\text{MTEC}}_{t, t + 1}^{s}$), meta-frontier Scale efficiency change (${\text{MSEC}}_{t, t + 1}^{s}$) and meta-frontier residual mix efficiency change (${\text{MRME}}_{t, t + 1}^{s}$).

#### FADN data used for TFP calculations

We use a farm-level data from the Polish Farm Accountancy Data Network (Polish FADN) for the years 2006–2015. The FADN sample consists of approximately 12 thousand^1^ farms per year, representing over 730^2^ thousand Polish farms with an annual standard output^3^ above 4000 EUR, and they provide 93% of total agricultural production in Poland (Floriańczyk et al. 2019). The sample is representative with respect to the type of farm, its economic size and localisation within 4 FADN regions and provides an appropriate base for comparisons among the farm types. A balanced panel has been built using farms which were recorded in the database during the analysed period and continuously belonged to the specified farm type. The panel consists of a full set of FADN observations (45,760) aggregated to 6 farming sectors: field crops (5840 observations), horticulture (1850), cattle farms (8050), granivores (2460), mixed farms (5680) and other farms (21,880)—see descriptive statistics in Table 5 in Appendix 1.

In our analyses, we used four types of inputs: (1) farm total utilised area in hectares (UAA) (FADN code SE025); (2) the labour force expressed in annual working units (AWU) (code SE010); (3) intermediate consumption in the Polish currency (PLN) (code SE275); and (4) capital in PLN (code SE436-SE446). As for the output, a single variable was used (also for the sake of the meta-frontier calculations), which is the value of the farm’s total output in PLN (code SE131). For the details on methodology of calculation the FADN variables, see (European Commission 2020).

The descriptive statistics (see Table 5) indicate that for farm size the smallest are horticulture farms (average of 9.7 ha) and the largest field crop farms (average of 67.7 ha). However, in terms of output produced the smallest farms are mixed farms (180,066 PLN) and the biggest are granivores farms (386,236 PLN). The labour use is the highest in horticulture farms (3.3 AWU) due to highly labour-intensive technology and the lowest in the mixed farms (1.8 AWU). Average intermediate consumption is the highest in granivores farms (254.957 PLN), while the lowest (being 3.5 times smaller) in mixed farms (72,808 PLN). Capital intensity is the highest in granivores (732,683 PLN), but the smallest in mixed farms (341,592 PLN). Therefore, the farm types obviously differ in terms of their single factor productivities—the highest land productivity is in horticulture farms but smallest in field crop farms; at the same time, however, labour productivity is the smallest in horticulture but the highest in granivores farms. Even this simple observation supports our approach to calculate total factor productivities (not partial or single factor productivities) to comprehensively assess the performance of different farm types in the analysed period of time.

Finally, the database was cleaned of outliers (as non-parametric analyses are sensitive to such observations) and restricted to positive values for all the variables. All the calculations presented in the results are indexed (so year 2006 = 1), and weighted (by number of representative farms) for each farm type outputs with the use of a geometric mean.

### Farm survey data

Our quantitative analysis is enriched by information from a large-scale survey carried out on a representative sample of 600 of the Polish farms included in the FADN database, which were interviewed in 2016. This subsample is driven from our FADN sample used for TFP calculations. The survey was particularly detailed with questions focusing on many details of farm practices, ecosystem-services and strategies by the five farm types at the centre of our interest (i.e. field crop farms, horticulture, granivores, cattle and mixed farms). For such farm types, we extracted information about their sources of innovation based on 4 categories and 13 indicators, as well as the adapted variety of ecosystem services—based on 4 categories and 17 indicators, as presented below:

(I) Sources of innovation:
  1. Gene revolution
    • Share of qualified seed material (where total consists of certified and non-certified)
    • Farmers’ opinion on GMO food-crops banned in the EU (share of farmers supporting ban)
    • Farmers’ opinion on GMO animal feed crops banned in EU (share of farmers supporting ban)
    • Farmers’ opinion on acceptable cost increases in the case of using GMO-free animal feed (an acceptable increase of GMO-free cost).
  2. Enhanced input delivery
    • Use of fertilisation plan as a tool for the decision of fertiliser doses (share of farmers).
    • Share of farms using chemical protection against:
      o diseases
      o pests
      o weeds
  (3) Hardware, software and data
    • Using soil testing (share of farms—soil tested in the last 5 years)
    • Soil test for mineral nitrogen content (share of farms)
    • Performing testing procedure of sprayer used for chemical protection (% share of farms)
  (4) Post-harvest management
    • Planting of catch crops (share of farms)
    • Cereal straw incorporation (share of farms)
(II) Ecosystem services:
  1. Biological pest control
    • Importance of following pest control methods (average: 0-meaningless, 6-very important):
      o biological protection,
      o mechanical protection,
      o selection of resistant varieties,
      o proper crop rotation.
  (2) Pollinator management
    • Presence on the farm:
      o Tree lines and bushes (share of farms)
      o Woodland on UAA (share of farms)
      o Permanently abandoned land (share of farms)
      o Hedgerows (share of farms)
    • Share of ecological areas (mentioned previously) in UAA (share of area)
  (3) Integrated crop-livestock practices
    • Application of animal manure on farm (share of farms)
    • Share of manured land in a single growing season (% UAA)
    • Time taken to incorporate manure into soil (average time in hours)
    • Presence of livestock (share of farms)
    • Stocking density (LU/ha – base on FADN data)
  (4) Rotation and soil conservation
    • Threat of soil erosion (share of farms)
    • Simplified and no-tillage crop cultivation
    • Taking biological aspects in crop rotation design (share of farms)

The statistics related to those indicators by farm type are presented in the next section. They are used for the assessment of the sources of technological change drivers in total factor productivity change among the groups of farms.

## Results

### TFP changes and drivers by farm types

**Fig. 3:**
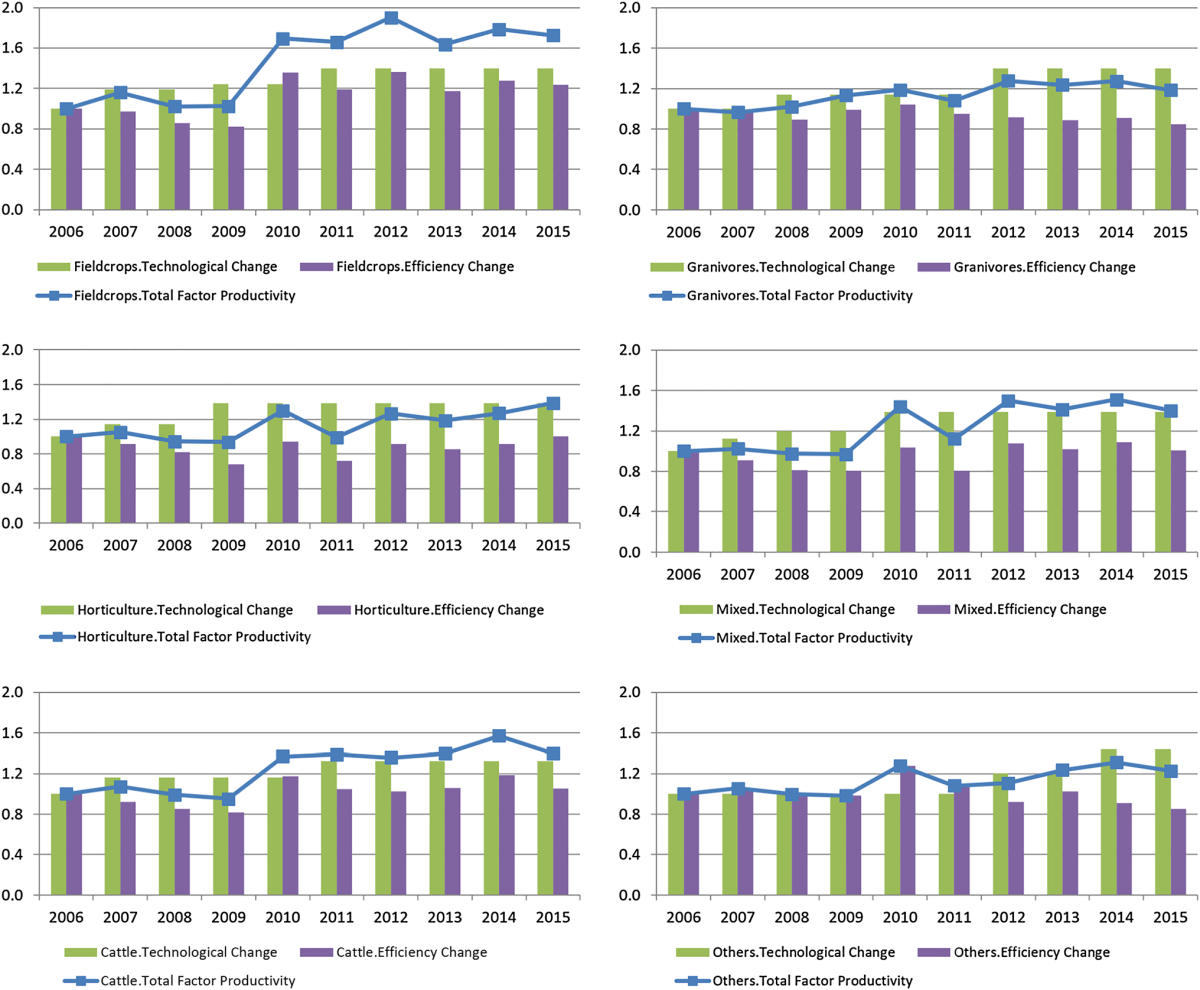
Färe-Primont productivity change and its decomposition, separate frontiers per farming sector. Source: Own calculations based on the polish FADN and Eq. (2) from Sect. Methods and data

The results obtained from the use of separate frontiers per farming type are presented in Fig. 3 and Table 6 in Appendix A. The indices of Färe-Primont productivity changes and its decomposition into technological change (TC) and efficiency change (EC) are interpreted so that an index above 1 means progress, equal to 1 means stagnation and below 1 means deterioration.

TFP for all farming sectors improved from 2006 to 2015. The highest TFP growth over that time was noted in field crop farms (72.5%) and the smallest in granivores farms (18.4%). Between them were the remaining sectors with the following TFP progress: in mixed farms by 40.1%, in cattle farms by 39.8%, in horticulture farms by 38.9% and in other farms by 22.9%. However, this was not a stable growth in TFP over that time as the farms were experiencing various adverse shocks and challenges. Two major adverse shocks affecting all farm types occurred in 2008 (global financial crises) and in 2014 (Russian embargo). The former shock affected the farms through: (1) a decline in the rate of economic growth, both in the country and abroad which affected volume of demand for agricultural products; (2) a fluctuating exchange rate which affected the “terms of trade” of the national economy and impacted agricultural trade; (3) the level of global crude oil prices which influenced the costs of production processes; and (4) increased levels of interest rates which caused difficulties in obtaining loans by farmers. The latter shock restricted volumes of traded agricultural products to Russia, which hit the meat and horticultural sectors in particular, for which the Russian market was a key one, so the situation pressured farmers to find new markets for those products. Besides these, the sectors experienced various sector specific shocks, both positive and negative.

In the year of the first crises, 2008, TFP patterns for most of the farm types either regress (horticulture, mixed and other farms) or slow down (field crops). Only granivores improved its TFP performance (by 5.6%). The pattern of recovering from the crisis in the year after was fairly similar for most of the sectors, i.e. by maintaining the similar TFP of 2009 to the previous year and substantial TFP recovery in 2010. The only outstanding sector was granivores in which TFP was already steadily growing in 2009 and 2010. After the second crises, the TFP slowed down from 2014 to 2015 in most farm types (the most in cattle farms by 17.5% and the least in field crop farms by 6%). The only exception was the horticultural farms which actually improved their TFP on average by 12%.

What is important from the point of view of the resilience analyses, as explained in our framework in Sect. 2, is not only the development of TFP but also its decomposition. Changes in its components over 2006–2015 reveal several interesting outcomes. First, for all the farm types, the majority of TFP growth was driven by technological change (TC indexes were higher than EC ones). Only in a few cases was the development the opposite, so that efficiency change drove the TFP growth. Such was the case of field crop farms, cattle farms and other farms in 2010 and also for the latter farms in 2011. This, therefore, is evidence of adaptation processes taking place in those farming sectors since their TFP change structure was adjusted. Second, in most farm types, the technological change went in the opposite direction to efficiency change, which is the case when not all producers are able to keep up in adjusting to new technologies (Dakpo et al. 2016; Latruffe et al. 2012; Brümmer et al. 2002). In most cases, technological change sped up, while efficiency slowed down or regressed. However, in some cases, the two components moved together, as in the case of mixed farms in 2009/2010, so after the recovery from the downfall of 2008 both technology change and efficiency change improved. We also observed several adaptation processes manifested by changes in TFP decomposition—change from technology driving efficiency which itself drives TFP change—in recovery from the first crisis (in the years 2010–2011) but not in the recovery from the second crisis (2014–2015) as the decomposition of TFP changes remained the same for all farming sectors.

**Fig. 4:**
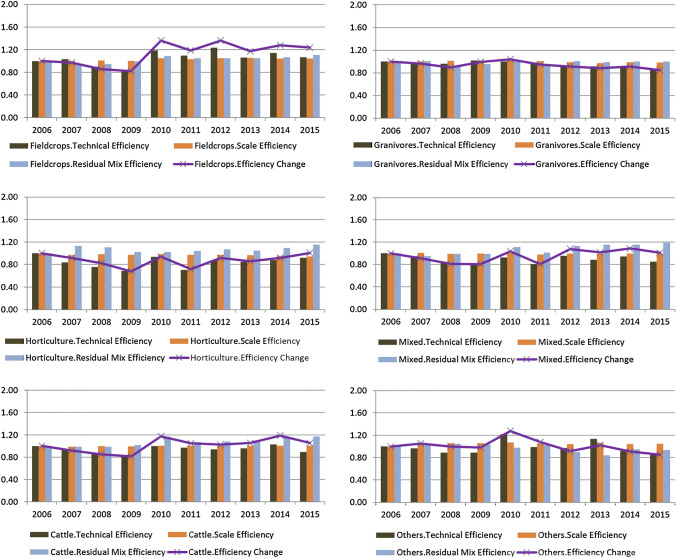
Efficiency change components evolution by farming sectors, for 2006–2015. Source: Own calculations based on the Polish FADN and Eq. (3) from Sect. 3

Further decomposition of the efficiency changes into technical efficiency change, scale efficiency change and residual mix efficiency change is presented in Table 6 in Appendix A and Fig. 4 in Appendix B. Over the years 2008–2009, the observed efficiency slowed or declined in all farming sectors, which was driven by technical efficiency changes slowing or regressing, while the other two types of efficiency—scale and residual mix—progressed or stayed the same. In 2010, however, the adaptation process occurred. Technical efficiency became the main driver of efficiency change (in field crop and other farm sectors) or was an equally strong driver as scale efficiency (in cattle and granivores farms) or at least became a more important driver in the structure of efficiency changes than before (in mixed farms and horticulture farms). In the years after the second crises 2014–2015, the technical efficiency deteriorated in all sectors (except horticulture) and the efficiency change was driven either by residual mix efficiency change (in horticulture, cattle and mixed farm sectors), or by scale efficiency (in other farm sector) or at least equally by scale and residual mix efficiency (in granivores). Only in field crop farms, despite slowing down technical efficiency, was it a main driver of total efficiency changes.

### Sources of technological changes

We show in Sect. TFP changes and drivers by farm types that technological change is a main driver of TFP change, so it is vital to investigate its sources and differences by farm type. Table 1 shows various innovations adopted by farming sectors, contributing to technological change, while Table 2 illustrates the ecosystem services adopted by farm types contributing to technological change. The farming sectors differ substantially in their adoption of innovations (grouped into categories proposed by Coomes et al. (2019), where the leading farm type was field crops with the highest percentage of farms adopting innovations, with granivores ranking second. The least innovating type seems to be mixed farms, see Table 1.

**Table 1:** Innovation-based sources of TFP’s technological change (% of farms)

	Unit	Field Crops	Cattle	Mixed	Horticulture	Granivores
*N* =	No. farms	157	131	215	41	56
Gene revolution	–					
GMO food-crops banned in EU	Share of supporters	72%	67%	73%	76%	61%
GMO animal feed crops banned in EU	Share of supporters	66%	64%	68%	76%	45%
Acceptable cost increase for GMO-free animal feed	Cost increase	10.8%	9.0%	11.1%	12.7%	8.4%
Use of certified seed material	Share of seeds	61%	55%	41%	70%	48%
Enhanced input delivery						
Use of fertilisation plan for setting fertiliser doses	Share of farmers	29%	9%	23%	2%	14%
Using an economic threshold to decide on using chemical protection:						
Against diseases	Share of farms	52%	47%	43%	47%	43%
Against pests	Share of farms	60%	50%	52%	53%	60%
Against weeds	Share of farms	55%	49%	49%	49%	57%
Hardware, software and data						
Using soil testing	Share of farms	66%	40%	46%	61%	55%
Soil test for mineral nitrogen content	Share of farms	22%	11%	13%	10%	7%
Certification of sprayer for chemical protection	Share of farms	87%	78%	82%	63%	82%
Post-harvest management						
Planting of catch crops	Share of farms	63%	53%	58%	17%	62.5%
Cereal straw incorporation	Share of farms	82%	12%	49%	27%	43%

Source: own calculations based on a survey of 600 Polish FADN farms

**Table 2:** Ecosystem services -based sources of TFP’s technological change (% of farms)

	Unit	Field Crops	Cattle	Mixed	Horticulture	Granivores
Biological pest control	No. farms	157	131	215	41	56
Importance of:	Scale 0 -meaningless, 6—very important					
Biological protection	Scale 0–6	1.03	1.21	1.34	1.78	1.06
Mechanical protection,	Scale 0–6	2.72	2.81	2.82	2.64	2.82
Selection resistant of varieties	Scale 0–6	2.68	3.07	3.00	2.13	2.89
Proper crop rotation	Scale 0–6	3.84	3.87	4.04	2.11	3.57
Pollinator management						
Presence on farm:						
Tree lines and Bushes	Share of farms	53%	48%	55%	32%	50%
Woodland on UAA	Share of farms	48%	47%	45%	34%	39%
Permanently abandoned land	Share of farms	41%	36%	29%	22%	29%
Hedgerows	Share of farms	13%	5%	9%	17%	9%
Share of scrubs in UAA	% UAA	0.52%	1.23%	0.70%	0.59%	3.52%
Integrated crop-livestock practices						
Presence of livestock	Share of farms	29%	100%	100%	12%	100%
Application of animal manure at farm	Share of farms	39%	97%	96%	34%	86%
Share of manured land in a single growing season	(% UAA)	6%	42%	30%	8%	34%
Time to incorporate manure into soil (average time in hours)	hours	12.26	14.76	12.57	15.45	13.46
Stocking density	LU/ha of UAA	0.04	1.02	0.81	0.11	3.44
Rotation and soil conservation						
Threat of soil erosion	share of farms	31%	36%	38%	17%	34%
Simplified and no-tillage cultivation	share of farms	6.55%	2.06%	3.08%	0.00%	1.88%
Considering biological aspects in crop rotation design	share of farms	65.6%	29.0%	32.4%	61.0%	32.1%

Source: own calculations based on a survey of 600 Polish FADN farms

Regarding gene revolution, among farmers specialising in crop production (field crops and horticulture) the strongest support for a GMO ban could be observed (up to 76%). These farmers also accepted the highest price increase (up to 12.7%) for GMO-free animal feed—the animal production can only be their minor activity so they prefer no risk options even at a higher price. This might suggest their negative attitude to gene innovations. However, such farmers buy the majority of seeds from certified sellers every year, which makes them the most sceptical to the introduction of new crop varieties. The animal farms (cattle and granivores) are not as strongly prejudiced against GMO crops (only 45% support a GMO ban for animal feed in granivores) and would like to spend less in the case of buying GMO-free feed (up to 9%). Mixed farms are buying the lowest share of certified seeds. This might indicate that field crops and horticulture farms are actively looking for innovations, however, farmers in this category are still strongly against the introduction of GMO crops, which is only one of the proxies for innovation applicability.

In the case of precise input delivery, field crop farms are noticeably the most innovative. Nearly, 1/3 of farmers prepare a fertilisation plan. Those farmers are the keenest to observe the level of the occurrence of pests and pathogens and use this information to make decisions on chemical plant protection. In this dimension, both horticulture farms and granivores could be placed in second place.

Additionally, in the case of using precise data for crop management, field crop farms are the leaders among all farm types. The majority of those farmers test soils, some of which even use quite advanced tests, assuming Polish conditions, for mineral nitrogen content in the soil and take care to obtain certification of sprayers. Regarding testing soil, which comes in second place, horticultural farms could be observed with only slightly lower results (61%). Farmers from granivores farms take care of the certification of sprayer for chemical protection almost as frequently as field crop farmers (82% of granivores farms versus 87% of crop farms)—see Table 1.

Regarding post-harvest management, field crops lead here too. Over 4/5 of straw is incorporated with the soil and catch crops are planted in 2/3 of farms. The share of farms growing catch crops in granivores is nearly as high as in the case of field crop farms. In the case of incorporating straw, the share of using this practice is lower in farms with animals (cattle, granivores, mixed) as the straw is used for animal production. In the case of horticultural farms, the lowest share of straw incorporation could be explained by a generally low share of cereals in cropping structure and using straw for soil cover in fruit production.

Generally, though the results show that Polish farmers on average are not strong innovators, it could be observed that field crop farmers are the keenest to use modern technologies and apply existing innovations in their daily practices.

The farming sectors substantially differ in their adoption of ecosystem services, although it is difficult to identify a leader—see Table 2. Depending on their specialisation, farmers seem to choose different services provided by the ecosystem. Regarding non-chemical plant protection methods, biological pest control is the most important in horticulture farms. In granivores farms, where crop production is of lower significance, the farmers appreciate the simplest mechanical means. In cattle farms, where mostly fodder crops are grown, the highest priority is to select the proper variety, and in mixed farms the proper crop rotation.

In the case of “pollinator management”, different kinds of shrubs (trees, woodland, hedgerows, etc.) are most frequently seen in crop farms, however, in general its share in UAA is the lowest. This could be the result of careful tillage practices aiming for efficient use of the main resource, which is the land. The biggest share of “pollinator-friendly” areas could be observed in granivores (3.53%). Often, crop production is treated in these farms as a marginal activity, thus the farmers are not trying to use every single plot of land for crop production.

As expected, the best integrated crop-livestock practices could be observed in the case of cattle farms. Cattle farmers use animal manure on their own fields to try to exploit its yielding potential. Even the granivore farms have a higher stocking rate, although not all of them use the manure on the farm. This is particularly the case in poultry farms, which usually have very low land resources and export all the manure from the farm. Farms specialising in crop production (field crops, horticulture) use animal manure to a much lower extent (39 and 34% of farms, respectively) as nearly all manure needs to be acquired outside of the farm, which is costly.

In the case of soil conservation, the crop specialised farms (horticulture, field crops) have the best situation regarding the threat of erosion. These farms usually operate on the better (heavier) soils compared with the animal farms. Again, field crop farms are leaders in the utilisation of the advantage of proper crop rotation (nearly 66% of farmers took biological aspects into consideration when choosing crops). This might be caused by a scarcity in organic matter due to limited manure application. A similar situation could be observed in horticulture farms (61%), while in other farms economic aspects are the most important when crop rotation is decided. Even the simplified and no-tillage cultivation methods are in general not very popular in Poland; field crops farms apply this technology to the greatest extent (in over 6.5% of farms). Due to the specificity of crops grown, this technology is not used in horticultural farms.

### Results with meta frontier and technology gap ratios

In Sect. TFP changes and drivers by farm types, we assessed the production frontier separately for each farm type, while here we combine all the farm types in order to see the leaders of the TFP changes and the existing technological gaps between the farm types (as explained in the methodological Sect. 3).

Table 7 presents the MFPP and its components for the Polish FADN farms together for the years 2006–2015. When all farms are taken together, we can see that the total farming sector experienced an increase of TFP by 27.8%, which was mostly driven by technological progress (53.2%), while efficiency actually fell (by 16.6%). As explained previously, the opposite direction of technological change and efficiency change can be explained by the fact that there are some farm types which do not cope immediately with technological advancements. Further decomposition of efficiency changes shows that it declined mainly due to technical efficiency decline (by 9.5%), followed by a decline in residual mix efficiency (by 7.2%) and scale efficiency (by 0.7%).

The meta-frontier approach allows the assessment of which farm types make up the frontier and which ones lag behind. The latter is indicated by TGR presented in Table 3 and TGR changes presented in Table 8 in Appendix A. The TGR ratios indicate that the meta-technology frontier was mostly comprised of the field crop farms as they had the highest average TGR among all sectors (0.88) and were located on the frontier five times (with TGR equal to 1) over the analysed period. This means they had access to the most productive technologies of all farming sectors. The second closest on average was the horticultural farming sector and other farm sectors, both with a TGR equal to 0.86. The least productive technology was seen in the case of mixed farms, with an average TGR of 0.65, while the sector was never on the frontier. This means that the sector on average reached only 65% of the maximum productivity, which is feasible under the meta-technology.

**Table 3:** Technology gap ratios for the Polish FADN farms, 2006–2015

	Field crops	Horticulture	Cattle	Granivores	Mixed	Other
2006	1.00	0.73	0.68	0.74	0.59	0.99
2007	1.00	0.86	0.71	0.55	0.59	0.74
2008	0.88	0.89	0.84	0.87	0.84	1.00
2009	0.82	1.00	0.53	0.61	0.51	0.66
2010	1.00	0.88	0.73	0.69	0.77	0.93
2011	1.00	0.85	0.81	0.64	0.61	0.70
2012	0.78	0.91	0.63	1.00	0.62	0.92
2013	0.87	1.00	0.75	0.89	0.68	0.95
2014	0.59	0.49	0.55	0.48	0.47	1.00
2015	0.88	1.00	0.68	0.66	0.82	0.71
Average	0.88	0.86	0.69	0.71	0.65	0.86

Source: Own calculations based on the Polish FADN and Eq. (5) from Sect. Methods and data

It is interesting to analyse the meta-frontier during the years around the two crises. During the first crisis, the frontier was made of the “other farms” sector in 2008, the horticultural farming sector in 2009 and the field crop farming sector in 2010. Interestingly, field crops led the frontier before the first crises (2006–2007) and again a few years later (2010–2011). During the second crisis, again other farms were leading in the immediate year of the crises in 2014, but horticultural farms took over in 2015.

The TGRC over the analysed period indicate that horticultural farms experienced the highest progress in their technological advancement over the period, namely by 18%, followed by mixed farms (by 10%) and cattle farms (by 2%). On average, the other farms sector is the one in which TGRC decreased the most, by 13%—see Table 8.

## Discussion of the results

In light of our framework (Sect. 2) and the results obtained through quantitative and qualitative analyses (Sect. 4), we can interpret the links between resilience and total factor productivity, as summarised in Table 4.

**Table 4:**
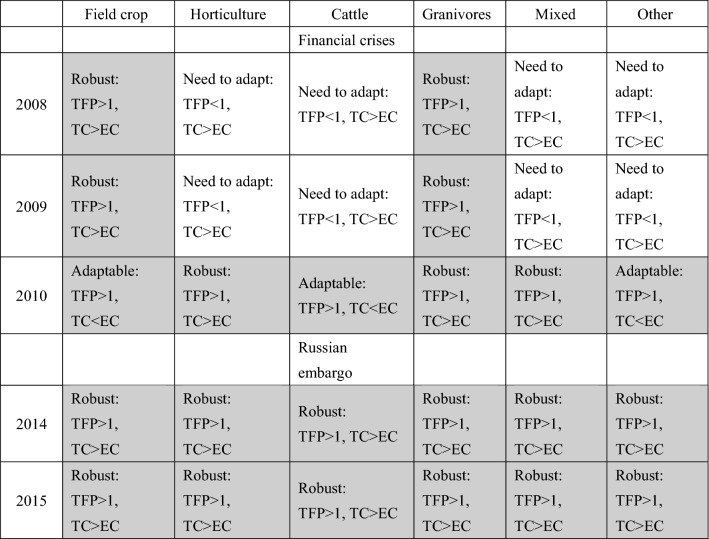
Assessment of resilience through TFP changes and its components changes TC and EC

Source: Authors’ own elaboration

*TFP* total factor productivity change, *TC* technological change, *EC* is efficiency change; values over 1 mean growth, values below 1 mean decline

The values of the FPP and its components (technological change (TC) and efficiency change (EC)) facilitated the assessment of the revealed resilience of the farming sectors to two major adverse shocks – the global financial crisis of 2008 and the Russian embargo of 2014. Table 4 revealed that in the case of a non-declining TFP (i.e. TFP equal to 1 or higher) we have two possibilities. First, the farming sector is robust if it manages to maintain its productivity (TFP > 1) despite no adjustments in its TFP decomposition—technology change drives TFP so it is larger than the efficiency change (i.e. TC > EC). Second, the farming sector is adaptable if it manages to maintain its productivity (TFP > 1) due to adjustments in its TFP decomposition—efficiency drives the TFP development (i.e. TC < EC). In the case of regressing TFP (TFP < 1), we also have two possibilities. First, if TFP declined as there was no substantial adjustments in TFP decomposition, it means that the sector failed to stay robust and needs to adapt (TC > EC). Second, if the TFP declined (TFP < 1) despite attempts to change its TPF decomposition (TC < EC), it means that it failed to adapt and needs to transform.

As we can observe in Table 4, the farming sectors differ in reactions to the shocks, among each other and in each of the two types of crises. In the first 2 years of the financial crisis (2008–2009), only field crop and granivores sectors stayed resilient, while the remaining sectors failed to do so and needed to adapt to regain the resilience revealed in TFP changes. Indeed, in the third year of the first crisis, 2010, all the sectors achieved either robustness (horticulture, granivores and mixed farming sectors) or adaptability (field crop, cattle and other farming sectors). In the case of the Russian embargo, the situation was different, as all sectors in both years (2014–2015) remained robust, even those that were the most affected in Poland, such as horticulture and meat (cattle and granivores). Interestingly, in none of the cases did any of the sectors experience a decline in their TFP while attempting to change their decomposition. There is, therefore, no situation when adaptation failed and the sector needed to transform. In fact, single farms in the FADN sample were changing the farming groups (so they individually transformed from one specialisation to another), so in a way transformation took place at the level of individual farms but not at the whole farming sector. We clearly observed in our result the responses of TFPs after the years of crises, however, for more formal links some authors suggest to apply a bias-corrected DEA analysis (Czyżewski et al. 2020b).

The explanation of differences in revealed resilience among the farm types obtained here can be partly explained by findings from other studies. The research on the resilience of the farming systems in the EU, including the Polish horticulture farming system, indicated that this sector’s main resilience capacity is robustness as it relies mostly on buffer resources (i.e. funds which build their reserves such as own savings and funds from Common Agricultural Policy), while it possesses relatively low-to-moderate abilities to adapt or transform. This was explained by deficiencies in resilience attributes such as low openness for cooperation among farmers, policy overregulation, problems with insurance policies and low policy responsiveness to the needs of that sector (Krupin et al. 2019). We can assess that the sector failed to be robust in the first two years of the first crisis, and only in the third year after the first crisis and throughout the second crisis did it regain its robustness.

Our result shows that the field crop sector lead in the Polish farming system in building up the technology frontier and in the adoption of innovation practices. At the same time, it was the only sector which managed to stay robust and adaptable over the two crises. The French studies carried out over a similar period (2002–2014) with the use of French FADN data and the same methods of Färe-Primont index also shows that this sector has access to the most productive technology and builds up the frontier (Dakpo et al. 2016). The shifts in the frontier in both countries are driven mainly by technological change so the sources of revealed resilience could be explained by insights into the drivers of those technological changes. In our survey, we investigated the sources of technological changes as suggested by Coomes et al. (2019a), divided into innovation and ecosystem services. However, in our survey, field crop farms were not leaders in their adoption. Additionally, more of the responding farms declared ecosystem services-based practices (in particular, biological pest controls, rotation and soil conservation) and few declared innovation-based technologies (in particular, the lack of precision technologies, as well as hardware, software and data use) in comparison to other farming types.

The importance of efficiency changes was smaller in the development of revealed resilience. This can partly be explained by studies demonstrating that technical efficiency is significantly affected by Common Agricultural Policy (CAP) subsidies, and the influence depends on the type of subsidies (Latruffe 2017; Latruffe and Desjeux 2016; Sckokai and Moro 2009; Czyżewski et al. 2017). Among our farm types, field crop farms are the beneficiaries of the highest per ha (decoupled) CAP subsidies, especially from Pillar 1 (i.e. direct payments for farmers), while the most productive in Poland were the CAP investment subsidies of Pillar 2 (i.e. Rural Development Programmes, so mainly investment subsidies or per ha payments for certain farming practices)—see Zawalińska et al. (2013). This could explain why more technological advancements (both innovation-based and ecosystem-based) were present in our survey in the case of cattle and granivores farms, which are the main beneficiaries of the CAP investment subsidies in Poland.

Interestingly, the farming sectors reacted differently to the two types of shocks. The farms were more resilient to the Russian embargo shock, although it had similarly severe economic implications as the global crisis. Even the most directly affected sector as horticulture managed to stay robust, although before the ban it exported up to 1/3 of its total production to Russia (out of which c.a. 60% were apples) (Guzek 2016). The sector maintained its TFP without adjusting its decomposition (between technological change and efficiency change). However, its efficiency component was altered—its residual mixed efficiency increased which can be explained by the fact that this sector relies least of all on CAP subsidies (small total area payments and no direct price support).

## Concluding remarks

The conceptual framework and empirical analysis presented in this paper provide the assessment of the specified “revealed resilience” manifested in total factor productivity changes and in its components in times of two major crises after Poland joined the EU, i.e. the global financial crisis of 2008 and the Russian embargo of 2014. The paper combined in one consistent framework the studies on the resilience of farming systems (drawing mainly from resilience capacities such as robustness, adaptability and transformability), studies on total factor productivity decomposition (identifying the relationship between its components such as technology change and various types of efficiency changes) and studies on the contemporary drivers of technological progress—namely innovation and ecosystem-based services. Therefore, the approach proved relevant to the specified (farming sector) “revealed resilience” (ex-post the shock) due to economic disturbances.

The paper transcends previous approaches in three aspects. First, it introduces a distinction between “potential” vs “revealed” resilience which helps to obtain a better understanding of the resilience performance. Second, it empirically assesses the proposed conceptual framework linking total factor productivity with resilience. Third, it shows resilience to specific shocks, economic and political one which also has strong economic consequences.

Our approach could be applied to gain a better understanding of farms’ responses to the current COVID-19 pandemic (see Meuwissen et al. 2021), as well as to design future CAP policy, which puts resilience among the priorities of the 2021–2027 period. We indicate that policy can firstly, foster “potential resilience” by supporting resilience capacities to strengthen resilience attributes, such as: diversity, flexibility, modularity, openness (as they help farming sectors to be more robust, adaptable and transformable). Second, the policy can monitor “revealed resilience” by including the total factor productivity measures for different eco-socio-economic farm types Po the Common Monitoring and Evaluation Framework. Besides, the policy makers can foster the research on links between “potential” and “revealed” resilience, that is how in practice those attributes actually result in total factor productivity changes and its components (through withstanding their performance without changes in its TFP decomposition, adapting by adjustment of TFP components or transforming into other sectors).

However, future research is needed in at least three directions. First, more farm sector specific studies on resilience capacities (robustness, adaptability and transformability) are needed to assess potential resilience and compare it with revealed resilience. Second, more studies on farm type specific drivers of total factor productivity (driven by technological and efficiency changes) are vital, including a variety of innovations, ecosystem services and beyond. Third, there is a need for more recent studies over a longer time frame, encountering more historical crises to analyse farm system responsiveness and also to the latest crisis, that of COVID-19, when data become available.

## Appendix A

See Tables 5, 6, 7, and 8.

**Table 5:** Descriptive statistics for the Polish FADN farms. 2006–2015

Type of farm; number of observations	Min	Max	Mean	Standard deviation	Coefficient of variation
Field crop farms; no. of observations: 5840					
UAA [ha]	5.0	731.7	67.7	79.9	1.2
Labour [AWU]	0.5	27.8	2.2	1.7	0.8
Intermediate consumption [PLN]	5965	2,727,074	117 345	154,458	1.3
Capital [PLN]	16,433	9,974,105	593,758	712,597	1.2
Total Output [PLN]	8773	5,373,387	233,837	302,517	1.3
Horticulture farms. no. of observations: 1850					
UAA [ha]	0.6	48.0	9.7	7.2	0.7
Labour [AWU]	0.5	20.2	3.3	2.4	0.7
Intermediate consumption [PLN]	4775	1,031,352	84 159	123,847	1.5
Capital [PLN]	12,650	6,837,266	555,720	686,535	1.2
Total output [PLN]	2078	1,970,760	200,713	231,375	1.2
Cattle farms; no. of observations: 8050					
UAA [ha]	2.6	197.4	30.8	20.8	0.7
Labour [AWU]	0.6	9.2	2.0	0.6	0.3
Intermediate consumption [PLN]	6032	1,400,221	90,024	92,643	1.0
Capital [PLN]	29,223	4,614,783	565,304	500,017	0.9
Total output [PLN]	6389	2,237,795	180,066	181,271	1.0
Granivores farms; no. of observations: 2460					
UAA [ha]	0.9	481.7	32.5	36.6	1.1
Labour [AWU]	0.5	16.9	2.0	0.9	0.4
Intermediate consumption [PLN]	14,453	3,517,470	254,957	298,764	1.2
Capital [PLN]	51,106	6,832,907	732,683	772,672	1.1
Total output [PLN]	17,438	6,078,128	386,236	447,616	1.2
Mixed farms; no. of observations: 5680					
UAA [ha]	1.9	699.2	27.6	34.2	1.2
Labour [AWU]	0.5	10.1	1.8	0.6	0.3
Intermediate consumption [PLN]	4151	1,421,352	72,808	73,381	1.0
Capital [PLN]	14,459	4,917,174	341,592	323,409	0.9
Total output [PLN]	5034	2,440,040	121,592	128,414	1.1
Other farms; no. of observations: 21,880					
UAA [ha]	1.1	485.2	32.2	33.8	1.0
Labour [AWU]	0.5	30.4	1.9	0.9	0.5
Intermediate consumption [PLN]	3896	1,546,628	89,391	112,596	1.3
Capital [PLN]	8360	5,194,883	398,086	410,894	1.0
Total output [PLN]	2330	2,335,045	151,630	184,315	1.2
All farm types together; no. of observations 45,760					
Total UAA [ha]	0.6	731.7	35.0	42.8	1.2
Total labour input [AWU]	0.5	30.4	2.0	1.1	0.5
Total Int. Cons. [PLN]	3896	3,517,470	99,700	134,918	1.4
Capital [PLN]	8360	9,974,105	469,823	515,449	1.1
Total output [PLN]	2078	6,078,128	177,992	228,376	1.3

**Table 6:** TFP changes and its component for the Polish FADN farms, using separate frontiers per farm type (normalised weighted results)

Years	TFP change (TFP)	Technological change (TC)	Efficiency change (EC)	Technical efficiency change (TEC)	Scale efficiency change (SEC)	Residual mix efficiency change (RMEC)
Field crop farms						
2006	1.000	1.000	1.000	1.000	1.000	1.000
2007	1.162	1.193	0.974	1.031	0.997	0.947
2008	1.022	1.193	0.857	0.898	1.009	0.946
2009	1.026	1.245	0.824	0.831	1.001	0.991
2010	1.695	1.245	1.361	1.191	1.048	1.091
2011	1.658	1.396	1.188	1.098	1.033	1.047
2012	1.901	1.396	1.362	1.233	1.051	1.050
2013	1.636	1.396	1.172	1.061	1.054	1.049
2014	1.785	1.396	1.279	1.144	1.045	1.070
2015	1.725	1.396	1.236	1.067	1.044	1.109
Horticulture farms						
2006	1.000	1.000	1.000	1.000	1.000	1.000
2007	1.051	1.144	0.918	0.840	0.968	1.130
2008	0.943	1.146	0.823	0.757	0.984	1.104
2009	0.939	1.382	0.679	0.683	0.971	1.024
2010	1.299	1.382	0.940	0.935	0.981	1.025
2011	0.991	1.382	0.717	0.705	0.975	1.044
2012	1.265	1.382	0.916	0.879	0.973	1.071
2013	1.184	1.382	0.857	0.847	0.964	1.049
2014	1.268	1.382	0.918	0.877	0.957	1.093
2015	1.389	1.382	1.005	0.922	0.944	1.155
Cattle farms						
2006	1.000	1.000	1.000	1.000	1.000	1.000
2007	1.073	1.164	0.922	0.944	0.990	0.986
2008	0.992	1.164	0.852	0.862	0.998	0.990
2009	0.951	1.164	0.817	0.810	0.994	1.015
2010	1.368	1.164	1.175	1.001	1.005	1.168
2011	1.388	1.324	1.048	0.972	1.006	1.073
2012	1.357	1.324	1.025	0.940	1.006	1.083
2013	1.398	1.324	1.056	0.957	1.005	1.098
2014	1.572	1.324	1.187	1.028	1.008	1.146
2015	1.398	1.324	1.056	0.895	1.008	1.170
Granivores farms						
2006	1.000	1.000	1.000	1.000	1.000	1.000
2007	0.966	1.000	0.966	0.961	1.000	1.004
2008	1.022	1.142	0.894	0.959	1.011	0.923
2009	1.134	1.142	0.993	1.021	1.017	0.955
2010	1.188	1.142	1.040	1.004	1.029	1.007
2011	1.082	1.142	0.947	0.986	1.007	0.953
2012	1.278	1.399	0.914	0.921	0.987	1.005
2013	1.237	1.399	0.885	0.917	0.974	0.991
2014	1.275	1.399	0.911	0.920	0.989	1.002
2015	1.184	1.399	0.846	0.860	0.982	1.002
Mixed farms						
2006	1.000	1.000	1.000	1.000	1.000	1.000
2007	1.022	1.122	0.911	0.952	1.006	0.951
2008	0.973	1.200	0.811	0.826	0.994	0.988
2009	0.968	1.200	0.807	0.817	0.997	0.991
2010	1.439	1.388	1.037	0.925	1.004	1.116
2011	1.120	1.388	0.807	0.811	0.982	1.013
2012	1.497	1.388	1.078	0.953	0.997	1.135
2013	1.411	1.388	1.016	0.884	0.994	1.157
2014	1.510	1.388	1.088	0.942	0.999	1.157
2015	1.401	1.388	1.009	0.853	0.995	1.189
Other farms						
2006	1.000	1.000	1.000	1.000	1.000	1.000
2007	1.054	1.000	1.054	0.970	1.064	1.021
2008	0.998	1.000	0.998	0.892	1.064	1.051
2009	0.983	1.000	0.983	0.891	1.063	1.039
2010	1.279	1.000	1.279	1.223	1.071	0.976
2011	1.080	1.000	1.080	0.991	1.054	1.033
2012	1.108	1.205	0.920	0.975	1.044	0.904
2013	1.236	1.205	1.026	1.138	1.068	0.844
2014	1.311	1.441	0.910	0.918	1.046	0.947
2015	1.227	1.441	0.851	0.866	1.049	0.938

Source: own calculations based on the Polish FADN and Eqs. (2) and (3) from Sect. Methods and data

**Table 7:** Average TFP changes and its component for the Polish FADN farms, using meta-frontier for all farm types

Years	TFP change (TFP)	Technological change (TC)	Efficiency change (EC)	Technical efficiency change (TEC)	Scale efficiency change (SEC)	Residual mix efficiency change (RMEC)
2006	1.000	1.000	1.000	1.000	1.000	1.000
2007	1.049	1.155	0.908	0.935	1.001	0.970
2008	0.985	1.155	0.853	0.876	1.004	0.970
2009	0.976	1.247	0.783	0.825	0.997	0.951
2010	1.313	1.247	1.053	1.179	1.005	0.889
2011	1.049	1.247	0.841	0.888	0.999	0.949
2012	1.112	1.247	0.892	0.902	0.993	0.996
2013	1.276	1.247	1.023	1.063	0.996	0.966
2014	1.362	1.532	0.889	0.959	0.993	0.933
2015	1.278	1.532	0.834	0.905	0.993	0.928

Source: own calculations based on the Polish FADN and Eq. (4) from Sect. Methods and data

**Table 8:** Technology gap ratios changes (TGRC) for the Polish FADN farms

	Field crops	Horticulture	Cattle	Granivores	Mix	Other
2006	1.00	1.00	1.00	1.00	1.00	1.00
2007	1.00	1.18	1.04	0.74	1.00	0.74
2008	0.88	1.22	1.23	1.17	1.43	1.01
2009	0.82	1.37	0.78	0.82	0.86	0.66
2010	1.00	1.20	1.07	0.93	1.31	0.94
2011	1.00	1.16	1.19	0.86	1.03	0.70
2012	0.78	1.25	0.93	1.34	1.06	0.92
2013	0.87	1.37	1.11	1.19	1.16	0.96
2014	0.59	0.67	0.80	0.65	0.79	1.01
2015	0.88	1.37	1.01	0.88	1.38	0.72
Average	0.88	1.18	1.02	0.96	1.10	0.87

Source: Own calculations based on the Polish FADN and Eq. (6) from Sect. Methods and data

## Appendix B

See Fig. 4.

## Footnotes

1 In 2015, it was exactly 12,313 farms.

2 In 2015, it was exactly 730.895 farms.

3 Standard Output is the average monetary value of the agricultural output at farm-gate price of each agricultural product (crop or livestock) in a given region.
